# Supplementary material for: Quantitative Visualization of Hypoxia and Proliferation Gradients Within Histological Tissue Sections
Source: Front Bioeng Biotechnol. 2019 Dec 5;7:397. doi: 10.3389/fbioe.2019.00397 (PMC6906162; doi:10.3389/fbioe.2019.00397)
Supplement: Supplementary file 1 [file Table_1.DOCX]

Supplementary Material


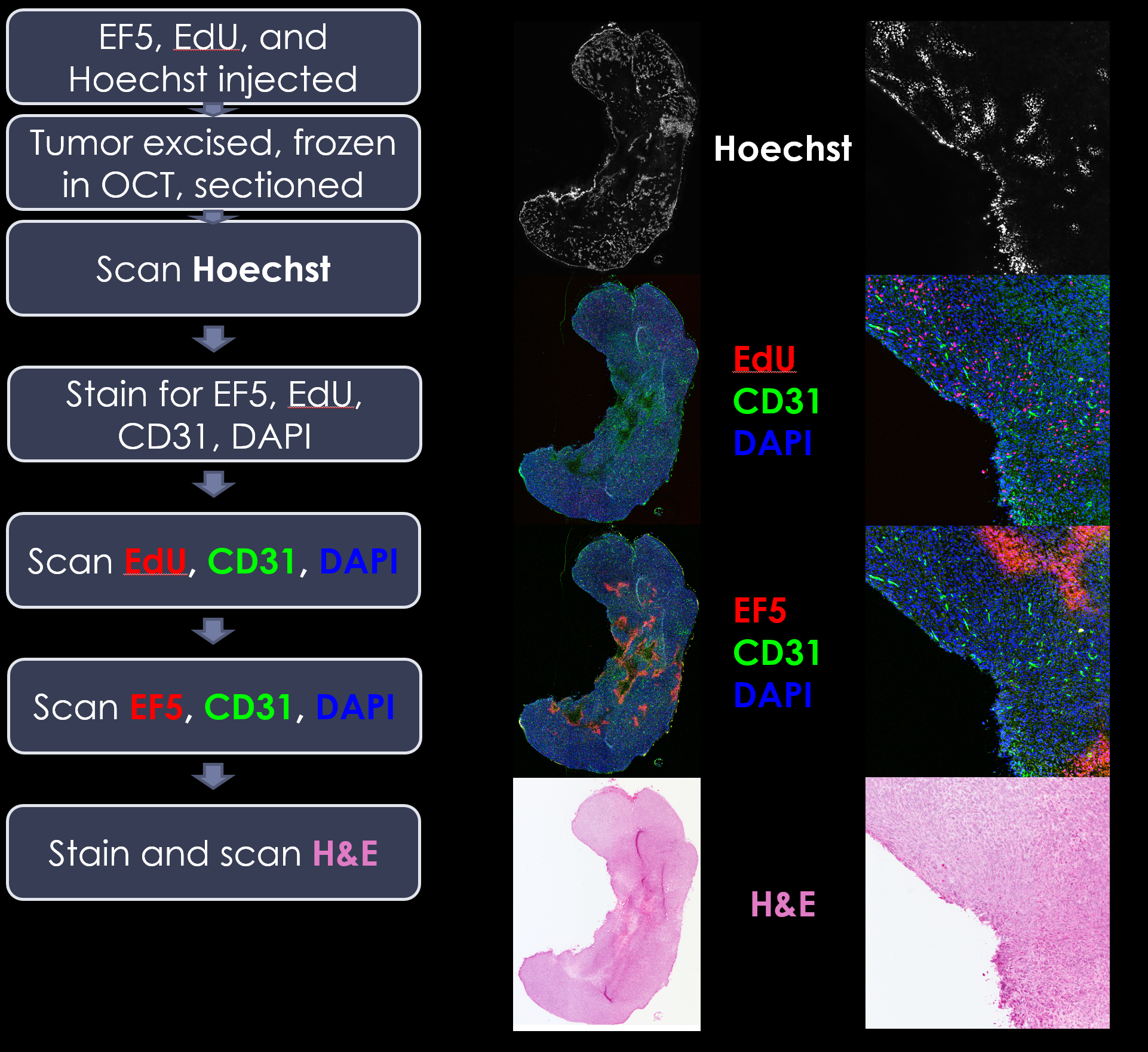


**Supplementary Figure 1.** **Immunofluorescent histology and imaging workflow.** Flowchart (left) depicting process of alternating scanning and staining needed to acquire the four images (right) utilized for subsequent image processing and analysis of perfused vessel distance, proliferation and hypoxia.
